# Bayou Hantavirus Cardiopulmonary Syndrome, Louisiana, USA, 2022–2023

**DOI:** 10.3201/eid3102.241069

**Published:** 2025-02

**Authors:** Emma Ortega, Sean Simonson, Elizabeth Shedroff, Shannon Whitmer, Amy Whitesell, Mary J. Choi, Trevor Shoemaker, Joel M. Montgomery, John D. Klena, Joseph Hennig, Theresa Sokol

**Affiliations:** Louisiana Department of Health, New Orleans, Louisiana, USA (E. Ortega, S. Simonson, T. Sokol); Centers for Disease Control and Prevention, Atlanta, Georgia, USA (E. Shedroff, S. Whitmer, A. Whitesell, M.J. Choi, T. Shoemaker, J.M. Montgomery, J.D. Klena); Louisiana State University, Lafayette, Louisiana, USA (J. Hennig)

**Keywords:** Bayou hantavirus, Bayou hantavirus cardiopulmonary syndrome, Orthohantavirus, Bunyaviridae, infection, genetics, transmission, reservoir, viruses, vector-borne infections, zoonoses, Louisiana, United States

## Abstract

During 2020–2023, we sequenced Bayou virus from 2 patients in Louisiana, USA, with hantavirus cardiopulmonary syndrome. Direct virus sequencing demonstrated an inferred evolutionary relationship to previous cases. Our findings demonstrate that separate virus spillovers cause isolated cases and probable wide distribution of Bayou hantavirus in rodents across Louisiana.

Hantavirus cardiopulmonary syndrome (HCPS) is a rodentborne zoonotic disease caused by infection with New World hantaviruses located predominantly in the Americas (*1*). HCPS is an acute febrile illness that can rapidly progress to acute respiratory distress syndrome and death; cases with fever but no respiratory involvement have also been identified (*2*). During 1993–2021, the hantavirus mortality rate in the United States was 35% (*3*). Disease is acquired after exposure to rodents, through inhalation of aerosolized virus from rodent urine and feces and, less frequently, from rodent bites (*4*). In the United States, 5 New World hantaviruses cause human disease (Table) (*5*,*6*).

**Table:** Most common New World hantaviruses known to cause hantavirus cardiopulmonary syndrome and the associated rodent reservoir, United States

Virus	Rodent reservoir
Bayou	Rice rat (*Oryzomys palustris*)
Black Creek Canal	Cotton rat (*Sigmodon hispidus*)
Monongahela	Deer mouse (*Peromyscus maniculatus*)
New York	White-footed mouse (*P. leucopus*)
Sin Nombre	Deer mouse (*P. maniculatus*)

*Oryzomys palustris* marsh rice rats are native to the southeastern United States, and Bayou virus–infected rodents have been identified in Texas, Louisiana, Georgia, and South Carolina (*7*,*8*). Only 7 human cases of Bayou virus infection have been reported, 5 in Texas and 2 in Louisiana, 1 of which was fatal (*2*,*4*,*5*). During 1993–2021, Louisiana reported 7 hantavirus cases; however, virus sequence information is available for only 1993 and 2013 cases (*4*,*6*). We describe 2 unrelated cases of Bayou HCPS from Louisiana reported in 2022–2023.

Patient 1 was a 66-year-old man with a medical history of tobacco use disorder who sought care at an emergency department after 4 days of chest pain, weakness, nausea, cough, and shortness of breath (*9*). Laboratory values indicated hemoconcentration, mildly elevated creatinine level, elevated lactate dehydrogenase level, and thrombocytopenia. Chest radiographs were concerning for bilateral infiltrates, and chest computed tomography (CT) showed small pleural effusions with patchy ground-glass opacities. At the time of arrival, the patient’s oxygen saturation was 91% with bilevel positive airway pressure and his oxygen requirements quickly escalated. His blood oxygen level decreased, and he was intubated. Laboratory analyses were notable for leukocytosis, worsening thrombocytopenia, and a granulocytic left shift; chest radiography showed worsening opacities. The patient had bilateral femoral artery clots and widespread petechiae, and he died 4 days after admission. Hantavirus serologic testing of samples collected before death were posthumously positive for IgM and negative for IgG. We could not obtain exposure information.

Patient 2 was a 56-year-old man with no relevant medical history. He experienced a syncopal episode preceded by a 1-week history of fever, cough, shortness of breath, malaise, diarrhea, and vomiting. At the time of arrival at the emergency department, he experienced a second syncopal episode. He had visited the emergency department once for this illness, which was diagnosed as a stomach virus. At admission, he was hypotensive with thrombocytopenia, leukocytosis, mildly elevated liver enzymes, elevated creatinine, and elevated lactate dehydrogenase level. Chest radiography was suggestive of bronchitis with pulmonary edema, and CT showed moderate interstitial pulmonary edema. Patient 2 was transferred to the intensive care unit for septic shock, complicated by thrombocytopenia, acute renal failure, and metabolic acidosis. Because his respiratory status deteriorated, bilevel positive airway pressure was administered, and metabolic encephalopathy developed. Subsequent CT showed bilateral pleural effusions and partial encapsulation of the left lower lung with left-sided pleural effusion. Thrombocytopenia worsened, and leukocytosis and creatinine level increased. Hemodialysis was started, and steroids and antimicrobial drugs were administered. The patient’s signs/symptoms gradually resolved, and he was discharged 15 days after admission. Hantavirus infection was confirmed by the presence of hantavirus-reactive IgM (IgG-negative) in a specimen collected 7 days after symptom onset; no subsequent specimens were collected. During a follow-up interview, the patient reported having cleaned out an uninhabited trailer during the 2–3 weeks before symptom onset, including tearing up carpets and insulation and working under the trailer without proper personal protective equipment.

Using the Altona hantavirus quantitative reverse transcription PCR (https://altona-diagnostics.com), we determined that virus cycle thresholds were 28.4 for patient 1 and 28.8 for patient 2. We generated complete Bayou virus large (L), medium (M), and small (S) segments directly from patient serum by using the RNA Exome Library Preparation unbiased sequencing method (Illumina, https://www.illumina.com) with pan-hantavirus enrichment oligonucleotides, followed by de novo assembly and reference mapping. L segment sequences from patients 1 (GenBank accession no. PP639088) and 2 (GenBank accession no. PP639090) were closely related to a rodentborne Bayou virus collected in 1996 (Figure, panel A). The M and S segments from both patients also clustered on Bayou virus-specific clades (Figure, panels B, C). M and S segments from both patients were more closely related to historic Bayou sequences than to each other; demonstrating that separate zoonotic spillovers probably caused disease (Figure, panels B, C). Patient 1 M and S segments (GenBank accession nos. PP639086 and PP639087) were related to Bayou sequences from *O. palustris* rats collected in 1996 from Galveston, Texas, whereas patient 2 M and S segments (GenBank accession nos. PP639091 and PP639089) were associated with a fatal Bayou virus case from northeastern Louisiana in 1993.

**Figure:**
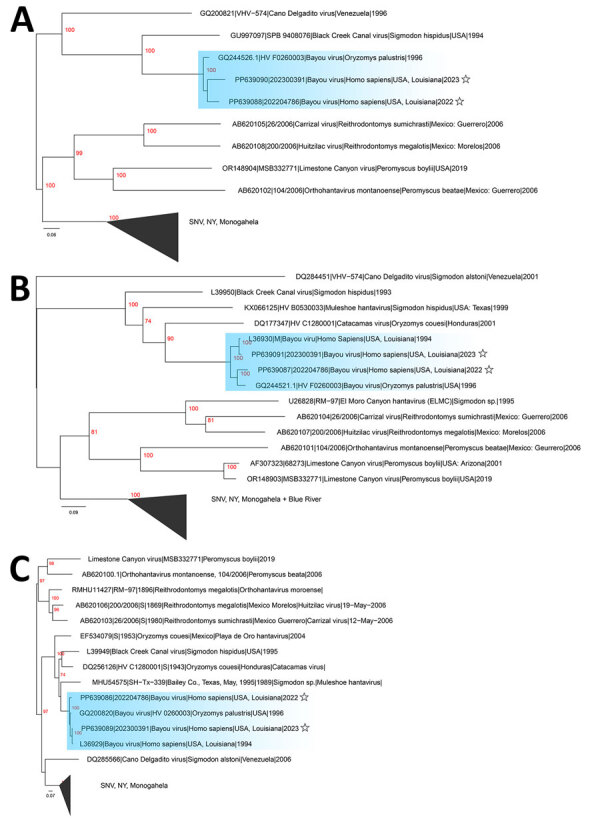
Inferred phylogenetic relationships between Bayou viruses from the United States and other New World orthohantaviruses. Trees were generated by maximum-likelihood using full-length large (A), medium (B), and small (C) segments. Major clades are collapsed and labeled. Trees are midpoint rooted with bootstrap support (n = 1,000 iterations) highlighted in red on each node. The Bayou virus clade is highlighted in blue. Stars indicate new sequences. Scale bars indicate nucleotide substitutions per site.

For both patients, hantavirus infection was initially exhibited by nonspecific signs/symptoms and quickly progressed to severe disease. Increasing surveillance efforts and clinician education, along with implementing the hantavirus 5-point screening tool, could improve rapid diagnostics during indistinguishable disease manifestation (*10*).
